# Danger in Tin Cans

**Published:** 1898-09

**Authors:** 


### Danger in Tin Cans.

Open a can of peaches, apricots, cherries, or other fruit—for all fruit is acidulous—let it stand for some time, and the fruit acids and the tin are ready to do their work of poisoning. A chemical knowledge that tells just how the dangerous compound is created is unnecessary to an avoidance of the peril. The rule to follow is never to make lemonade or other acidulated drinks in a tin bucket, nor allow them to stand in a vessel of tin; and in the case of canned fruits or fish, immediatetly upon opening the can turn the contents out upon an earthenware plate, or into a dish that is made of earthenware or glass.

Fruits in hermetically sealed cans, if properly prepared, generate no poison. As soon as opened the action of the acid on the tin, with the aid of the atmosphere, begins, and in a short time the result is a deadly poison. This brief treatment of the question should be remembered by everyone, and its instructions followed. The general press also should aid in disseminating this simple knowledge.—*Popular Science.*

